# Intra-prostatic gold fiducial marker insertion for image-guided radiotherapy (IGRT): five-year experience on 795 patients

**DOI:** 10.1186/s12880-023-01036-z

**Published:** 2023-06-12

**Authors:** Ali Mahdavi, Bahram Mofid, Farzad Taghizadeh-Hesary

**Affiliations:** 1 Department of Radiology, Imam Hossein Educational Hospital, Shahid Beheshti University of Medical Sciences, Tehran, Iran; 2 Department of Radiation Oncology, Faculty of Medicine, Shahid Beheshti University of Medical Sciences, Tehran, 1985717443 Iran; 3 ENT and Head and Neck Research Center and Department, The Five Senses Health Institute, School of Medicine, Iran University of Medical Sciences, Tehran, Iran; 4 Department of Radiation Oncology, Iran University of Medical Sciences, Tehran, Iran

**Keywords:** Adverse effects, Efficacy, Gold fiducial marker, Image-guided radiotherapy, Prostate cancer

## Abstract

**Introduction:**

Prostate cancer is the second most commonly diagnosed cancer in males. The use of intra-prostatic fiducial markers (FM) for image-guided radiotherapy (IGRT) has become widespread due to their accuracy, relatively safe use, low cost, and reproducibility. FM provides a tool to monitor prostate position and volume changes. Many studies reported low to moderate rates of complications following FM implantation. In the current study, we present our five years’ experience regarding the insertion technique, technical success, and rates of complication and migration of intraprostatic insertion of FM gold marker.

**Methods:**

From January 2018 to January 2023, 795 patients with prostate cancer candidate for IGRT (with or without a history of radical prostatectomy) enrolled in this study. We used three fiducial markers (3*0.6 mm) inserted through an 18-gauge Chiba needle under transrectal ultrasonography (TRUS) guidance. The patients were observed for complications up to seven days after the procedure. Besides, the rate of marker migration was recorded.

**Results:**

All procedures were completed successfully, and all patients tolerated the procedure well with minimal discomfort. The rate of sepsis after the procedure was 1%, and transient urinary obstruction was 1.6%. Only two patients experienced marker migration shortly after insertion, and no fiducial migration was reported throughout radiotherapy. No other major complication was recorded.

**Discussion:**

TRUS-guided intraprostatic FM implantation is technically feasible, safe, and well-tolerated in most patients. The FM migration can seldom occur, with negligible effects. This study can provide convincing evidence that TRUS-guided intra-prostatic FM insertion is an appropriate choice for IGRT.

## Introduction

Prostate cancer is the second most commonly diagnosed cancer in males [1]. According to patient preferences and life expectancy, treatment options include active surveillance, radical prostatectomy, or radiotherapy (external beam radiation therapy [EBRT] or brachytherapy [2, 3]. Regarding EBRT, daily gland displacement could lead to a target missing secondary to significant prostate motion during radiotherapy [4]. Therefore, prostate motions must be considered to set the target margins for radiotherapy [5]. Fiducial markers (FMs) can facilitate the tracking of inter- or intrafraction prostate motions, thereby, image-guided radiotherapy (IGRT). Hence, radiotherapy with FMs benefits from reduced target margins [6]. Besides, FMs can be applied for daily prostate position verification and correction before and during IGRT [7]. The accuracy, safety, low cost, and reproducibility of FM have made it the most acceptable approach to tracking prostate motions during radiotherapy [8]. The hydrogel spacer method is another way to reduce the radiation that reaches the organ at risk (OAR). Pepe et al. found that using a hydrogel spacer before hypofractionated prostate cancer radiotherapy helps to reduce the genitourinary and rectal toxicities [9].

There are two main approaches for FM insertion: transrectal and transperineal, both usually under trans-rectal ultrasound (TRUS) guidance. Both approaches are safe and well tolerated [10–13]. The transperineal approach has a lower risk of infection rates. Nevertheless, this difference is minimal (0.5%) [14]. On the other hand, the transperineal fiducial marker implantation may lead to more risk of bleeding and pain [15]. Overall, both approaches are acceptable in nowadays practice, and using transrectal or transperineal approach are mainly based on radiologist preference. Several studies reported low or moderate rates of complications following FM [11, 12, 16, 17]. However, controversies also exist regarding complication rates; Loh et al. realized that the adverse effects of FM implantation are underestimated [15].

The efficacy of FMs for prostate IGRT is based on the assumption that each marker will remain fixed in position during planning and treatment. FM migration can occur; however, the migration rate and its importance in clinical practice, which could lead to significant limitations to utilizing FMs for prostate radiotherapy, should be assessed. Also, developing a standardized protocol for FM insertion to minimize known complications and migration should be considered.

In the current study, we present our five-year experiences of TR insertion of intra-prostatic gold FM in 795 patients with prostate cancer candidates for IGRT. The detailed procedure and rates of complication and migration are outlined. To the authors’ knowledge, this is the largest series of intra-prostatic FM insertion. In addition, this is one of the published reports of FM insertion in patients after radical prostatectomy. The results of this study can provide a basis for future studies on the application of intraprostatic FM for advanced radiotherapy techniques.

## Materials and methods

### Study design and endpoint

**Fig. 1:**
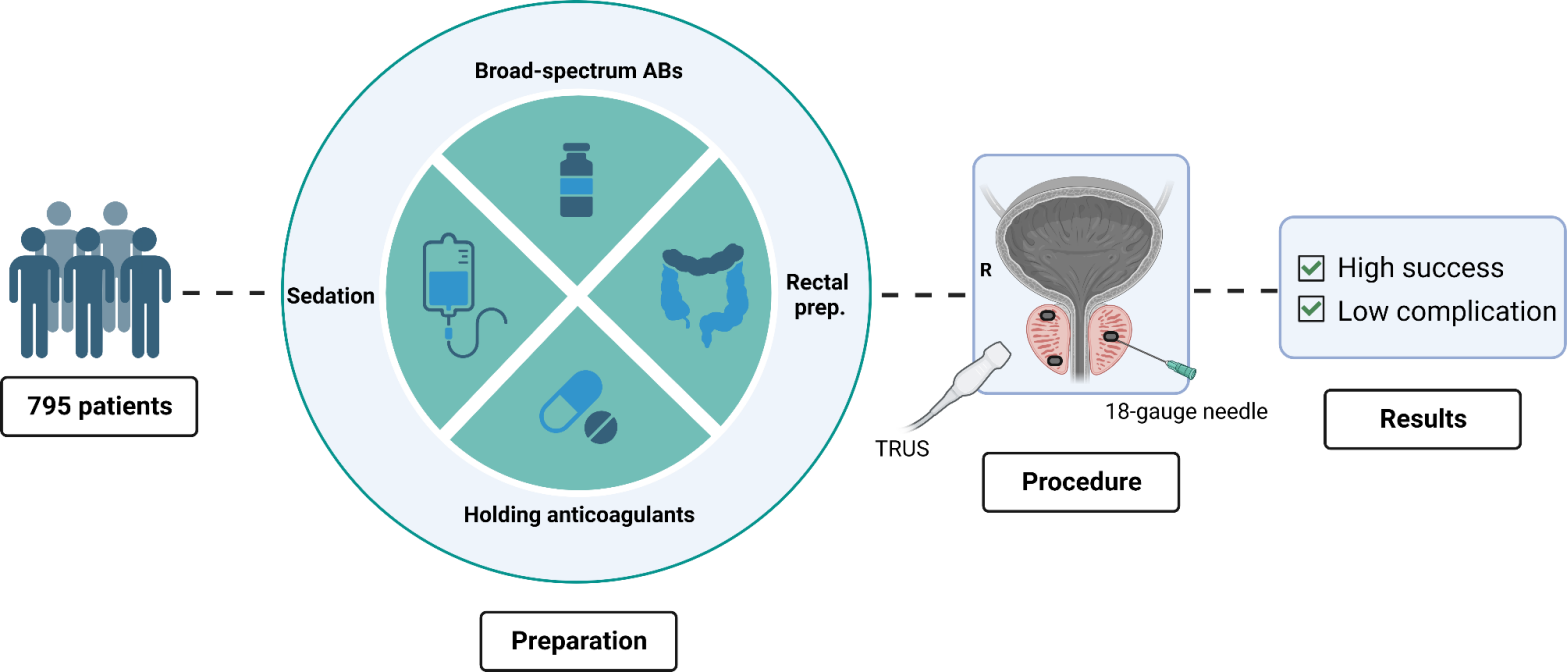
The study summary demonstrating the patients’ preparation, applied technique for fiducial marker insertion, and outcomes

This is a prospective cohort of patients with prostate cancer who underwent FM insertion to facilitate pelvic IGRT. The primary endpoint of this study is to report the success and complication rates of our experience and compare them with similar studies in the literature. Figure 1 denotes the patients’ preparation, applied technique, and the outcomes.

### Preparation

The patients were asked for a history of drug allergy, and if there were no major allergic drug reactions, prophylactic antibiotics (including ciprofloxacin 500 mg BID and metronidazole 250 mg TDS) were orally started 48 h before the procedure and extended up to 48 h thereafter. For patients with a higher risk of infection (based on clinical history), another 40 mg intramuscular dose of gentamycin was also administered on the day of the procedure. Bowel preparation included a clear liquid diet starting the day before the procedure.

Anticoagulation and antiplatelet medications were withheld 48 h before the procedures (if possible). Laboratory coagulation tests (PT, INR, and platelet count) were not routinely checked. However, in high-risk patients (patients with a recent major hemorrhage or prosthetic valve replacement), coagulation tests were performed the day before the procedure, and if there was a significant abnormality (INR > 1.7 or platelet < 50 000), the procedure was withheld until the coagulation indices became normal.

Patients underwent moderate sedation using 1–2 mg IV midazolam diluted in normal saline. Just five minutes before the fiducial insertion, bowel preparation (with 25 cc rectal enemas containing lidocaine gel combined with povidone-iodine) was performed. After marker insertion, all the patients were observed for 6 h in a recovery room.

### Technique

TRUS was performed using an endorectal ultrasound probe (Affinity 70, Philips) with the patient in the left lateral decubitus knee-chest position. A plastic needle holder was installed beyond the ultrasound probe for precise needle location. First, an ultrasound examination of the prostate gland was performed. For patients with no history of radical prostatectomy, the prostate gland was assessed for any suspicious hypoechoic nodule, especially in the peripheral gland, and for a possible extraprostatic extension. The prostate base was identified by the seminal vesicles located at the posterior surface of the gland bilaterally, whereas the apex was demarcated inferiorly by the midline urethra, exiting the prostate anteriorly and inferiorly. Regarding postoperative status after radical prostatectomy, we waited at least four months before gold marker insertion; however, if any hematoma were present on ultrasound examination before marker insertion, the procedure withheld for another month. In this case, the surgical bed was assessed for possible collection or hematoma, and in patients with remote surgery, urethral anastomotic sites and bladder base were also assessed carefully for a possible recurrence. For patients with a previous transurethral resection of the prostate (TURP), in which the central gland is removed for benign prostatic hyperplasia (BPH) treatment, the peripheral gland was assessed for evidence of any suspicious hypo-echoic lesion.

**Fig. 2:**
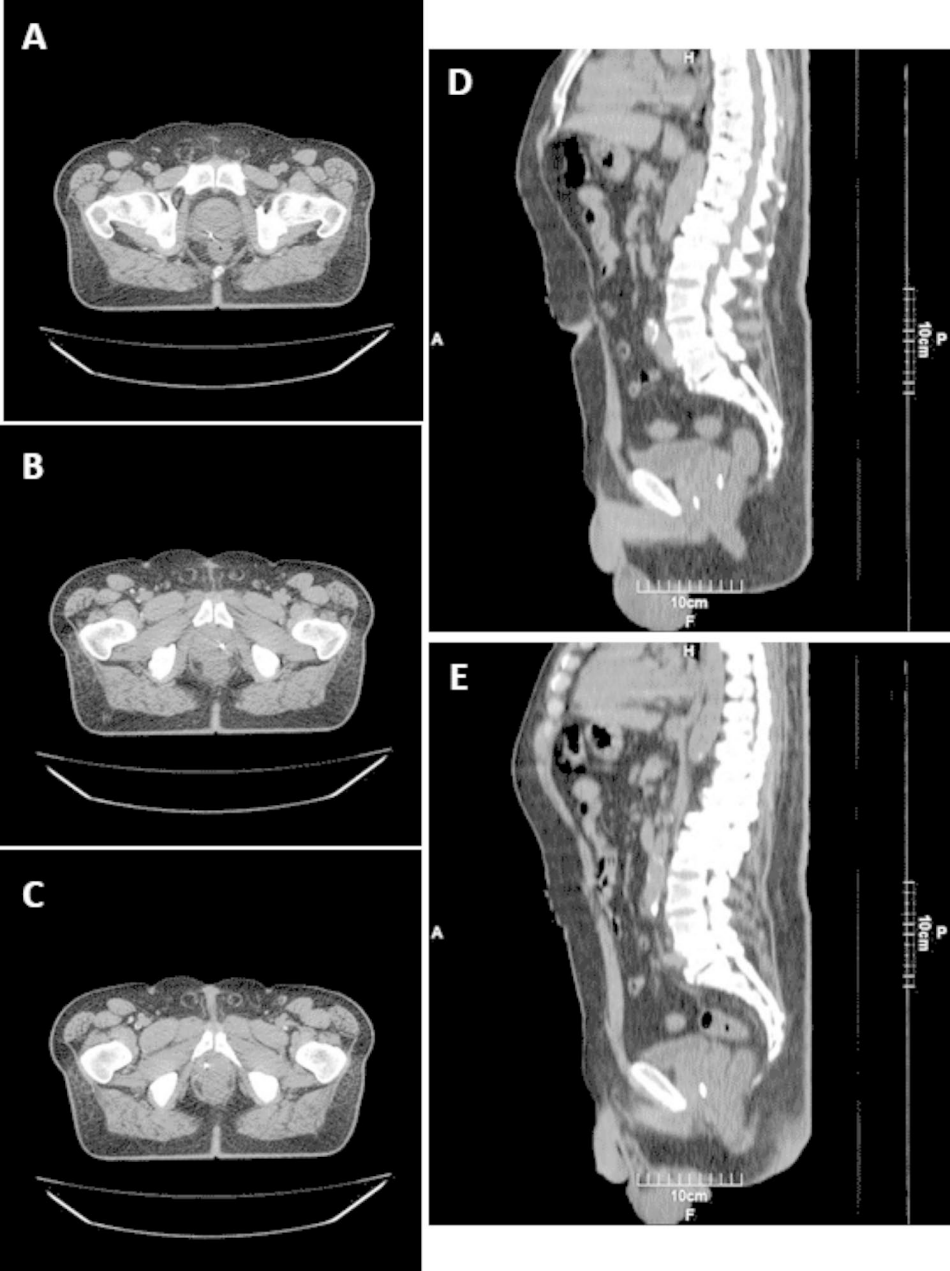
Fiducial gold marker insertion in a patient with confirmed prostatic adenocarcinoma candidate for IGRT. Three fiducial markers were inserted in different parts of the prostate gland under TRUS guidance, and the position was confirmed with a CT scan performed for planning. Two fiducial markers were placed close to the prostatic lesions (white arrows in Fig. 3)

**Fig. 3:**
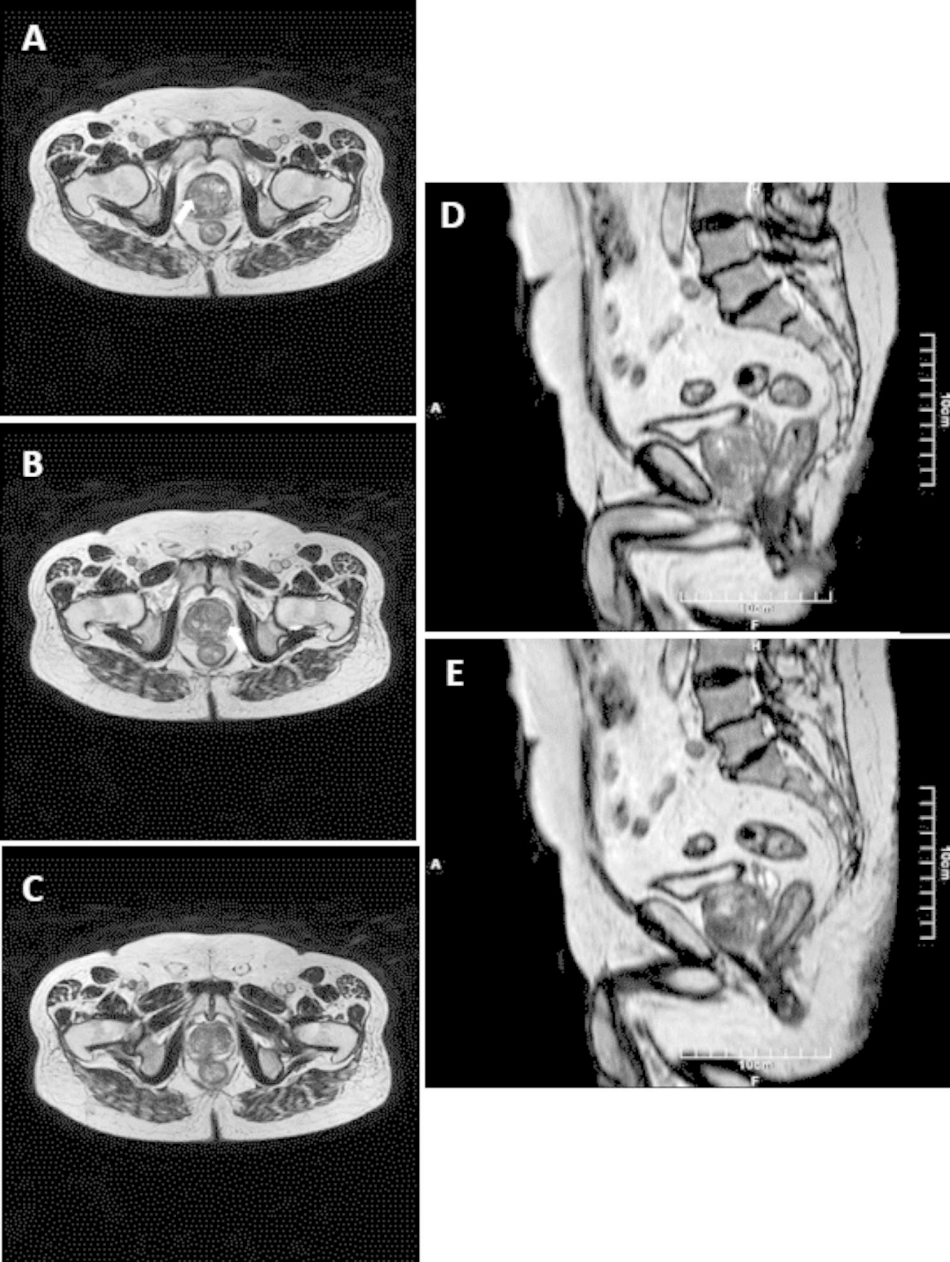
In MR imaging, which was also performed for planning, no obvious signal disruption was present due to the non-magnetic property of gold markers (same patient as Fig. 2). White arrows demonstrate the prostatic lesions

Three fiducial markers (3 mm * 0.6 mm) were inserted using an 18-gauge Chiba needle. In patients without a history of radical prostatectomy, the markers were inserted in three parts of the prostate gland: the right base of the peripheral zone, the left mid-gland peripheral zone, and the right apex of the peripheral zone. In patients with a history of TURP same sites were selected. In patients with radical prostatectomy, the prostate’s normal anatomy is absent. We inserted two markers at the level of vesicourethral anastomosis on each side, and the third marker was inserted on the right side of the bladder neck. These areas are selected because these locations are the most common site of local recurrence based on previous studies [18, 19]. Figures 2 and 3 represent the CT and MR images of FM locations in a patient with confirmed prostatic adenocarcinoma candidate for IGRT. If any suspicious hypoechoic nodule especially in peripheral gland was found on ultrasound examination, at least one of the markers was inserted as close as possible to the lesion.

### Follow-up

Fiducial positioning was confirmed with a post-procedure pelvic radiograph and a simulation CT scan within seven days after marker implantation for radiation treatment planning. Also, patients underwent MRI for planning. Due to the non-magnetic property of gold markers, no metallic artifacts were observed in MR images making these markers suitable for prostatic IGRT. Patients underwent radiation treatment over six to seven weeks using intensity-modulated radiotherapy (IMRT) techniques. The planned radiation doses to the primary site were 70 Gy in 35 fractions (after radical prostatectomy) and 70 Gy in 28 fractions (for definitive treatment).

### Ethical issues

Informed consent was taken from the patients before the procedure. Every patient and his associates were educated about minor complications (such as subtle hematuria or hematochezia just after the procedure or low-grade fever). Also, they were informed about warning signs (e.g., fever of more than 38.5 ^o^C, urinary obstruction, severe hematuria, or hematochezia). The patients were observed for major complications up to 7 days after the procedure, and they were asked to go to the emergency ward if their warning clinical symptoms persisted.

All experimental protocols were carried out in accordance with relevant guidelines and regulations and approved by the Institutional Review Board of Shahid Beheshti University of Medical Sciences (SBMU). This study was performed in line with the principles of the Declaration of Helsinki. Being an anonymous analysis of clinical outcomes of patients treated as per institutional protocol, additional ethical clearance was waived by the Institutional Review Board. The reporting of this prospective study follows the STROBE checklist for cohort studies (available at: https://www.strobe-statement.org/checklists/).

### Statistical analysis

Categorical variables are summarized as numbers and percentages and were compared using the Chi-Square test. Continuous variables are summarized using mean and standard deviation. All tests were two-sided, and the statistical significance was set to 0.05. We used IBM SPSS Statistics® (ver. 26) for statistical analysis.

## Results

### Patients’ characteristics

From January 2018 to January 2023, 795 patients enrolled in this study. The patients’ ages ranged between 47 and 94 years, with mean age of 70.3 (± 9.1) years. Most patients (532 cases, 66.9%) had no history of radical prostatectomy. Among the remaining cases, 64 patients (64/263, 24.3%) had a remote history of TURP for BPH disease. In the radical prostatectomy group, 109 patients (41.4%) had only local recurrence confirmed with pelvic MRI or PET scan findings.

### Intraprocedural complications

All procedures were completed well, and all FMs were inserted in the designated locations successfully. All patients tolerated the procedure well with minimal discomfort. None of the procedures were canceled due to excessive discomforts, such as severe anus contraction, anus pain, or excessive anxiety. The pain and distress levels were acceptable, and no one complained of prolonged anal pain after the procedure.

### Late complications

During the follow up, 8 patients (1%) returned with sepsis symptoms (fever more than 38.5 ^o^C, severe chill, and myalgia) unresponsive to conventional OTC drugs and administered oral antibiotics. This rate was nonsignificantly higher in patients with a history of radical prostatectomy (6 vs. 2, *P* = 0.6). Among them, seven patients were treated with intravenous (IV) antibiotics at the emergency department without need to a prolonged admission and were discharged within 12 h. The remaining one patient was discharged after two days of antibiotic therapy.

Among the overall cohort, 13 patients (1.6%) complained of intermittent urinary obstruction within 24 h after the procedure and were managed successfully using urinary catheterization. There was no reported major urinary or rectal bleeding, which continued and required hospitalization. Although mild hematuria and hematochezia a few hours after the procedure were relatively common, almost all were resolved shortly. No other major complication was reported.

### Marker migration

Marker migration through the rectal wall into stool was detected in two patients (0.2%), both with a history of radical prostatectomy. No marker migration through the venous plexus was detected. Marker positions were verified daily using an electronic portal imaging device (EPID). The acceptable limit of FM migration was set to 2 mm. No significant fiducial migration was detected throughout radiotherapy.

## Discussion

Advanced radiotherapy techniques, such as IMRT, can lead to superior precision and optimization of the treatments associated with reduced doses to the OAR, thereby toxicities. This benefit can help to escalate the dose to the target tissue and improves the disease control. The major problem in IMRT of prostate cancer is the considerable prostate motion. It has been demonstrated that the prostate can displace even 18 mm during a radiotherapy session [20]. This issue would increase the target volume and lead the protecting the OAR (including rectum and bladder) into trouble [21]. This problem can be minimized by tracking the prostate motion, developed in IGRT systems [22]. To this end, one of the most common approaches is FM tracking during radiotherapy sessions.

Evidence supports the FM insertion to track prostate motion during the radiotherapy course. It can assist to improve the biochemical tumor control [23] and reducing the toxicities [24, 25]. However, the available information on its success rate and complications is not in concert. Moman et al. reported one of the largest published series about the practical feasibility, success, and complication of intraprostatic FMs. They found a success rate of 99% and complications rate of 3.9%. The most common reported complication was hematuria (1%) [14]. Igdem et al. prospectively quantified patient-reported morbidity of TRUS-guided TR implantation of three gold FMs in 135 respondents. No anesthesia was applied. Five patients reported rectal bleeding and 20 patients experienced hematuria. No case required additional therapeutic intervention. Three patients experienced urinary infection requiring additional antibiotics [11]. In another study by Kably et al. on 75 patients, the success and complication rates were 99% and 10.6%, respectively. The most common complication was intraprostatic hemorrhage (4%) [16]. Our study reports the same endpoints in 795 patients, which constitute one of the largest series. In this cohort, the success rate was higher (99.8%), and the complication rate was lower (2.6%). Grade 3–4 complications was not detected and the reported complications (2.6%) were mild sepsis or urinary retention. Lower complications may reside in our technique. Applying needles with a narrower lumen compared to the Kably et al. (gauge 18 vs. 17) may justify the lower rates of urinary retention (1.6% vs. 11.4%) and perirectal hemorrhage (0 vs. 1.3%). Also, the cessation of anticoagulant/antiplatelet medication two days before the procedure can contribute to the latter finding. Linden et al. reported no immediate complications, including urinary retention or gross hematuria in 98 patients [12]. Linden’s and ours approach were in common in terms of the applied needle size (18-gauge). Therefore, using 18-gauge needle can help to reduce the immediate complications. Linden employed a triangular arrangement of markers ( at right base, left mid-gland or base and right apex) similar to our approach [12]. Shinohara and Roach also highlighted the importance of avoiding of the urethra to ensure markers are not subsequently lost to voiding [13].

Literature review reflects the controversy in the post-procedural infection rate. Several studies have reported low infection rates (around 2%) [16, 26]; while another study reported higher rates (7.7%) [15]. In the current study, the infection rate was 1%. Compared with the other studies, lower symptomatic infections may root in the applied broad-spectrum antibiotics and the addition of povidone-iodine to the rectal enema just before the procedure. Prophylactic antibiotic is essential in the transrectal approach. However, the optimal antibiotic regimen is not determined. We utilized a combination of ciprofloxacin, an available broad-spectrum bactericide, and metronidazole, which has good coverage on gram-negative bacteria. Most of our patients tolerated the antibiotic regimen well, but three cases ceased metronidazole because of subtle gastrointestinal discomforts. Povidone-iodine seems to be an important factor in diminishing the sepsis rate by reducing rectal bacterial flora.

The optimal anesthesia for FM placement is a matter of debate [16]. This study demonstrated the tolerability of the procedure with sedation using 1–2 mg midazolam. Replacing local anesthetics with general anesthesia can contribute to the reduction in infection rates. The slow injection of IV midazolam, under cardiac monitoring, effectively reduces procedural anxiety and increases patient satisfaction.

Numerous studies have been carried out to identify the factors that predict complications in prostatic FM placement. Kably et al. found that advanced T-stage and metastatic status were the predictive factors, while patients’ age and PSA levels did not play a significant role [16]. However, Igdem et al. discovered that T-stage did not lead to an increase in bleeding complications [11]. This disagreement may be due to small sample sizes and diverse patient populations.

Reports on FM numbers and sites are diverse. Many authors have reported studies using three or four implanted gold markers [6, 27–30]; on the other hand, there are reports using two elongated markers placed at either side of the prostate to localize the prostate for IGRT [31]. Using three markers seems an acceptable approach to monitor prostate position and volume changes that can occur over time owing to hormone or radiation therapy [32]. Previous studies showed that FM migration within the prostate during radiotherapy is negligible [33, 34]. We employed three FMs placed in a 3D triangular arrangement to facilitate assessment in the three cardinal directions. In this cohort, FM migration was rare and occurred in 2 out of 795 patients (0.2%). It is worth noting that both cases of marker migration occurred in the prostatectomy group shortly after insertion. In both cases, the migrated marker was one of the vesicourethral markers. Therefore, this migration did not clinically influence treatment planning, as the remaining marker adjacent to the vesicourethral junction was deemed sufficient for planning purposes. The higher likelihood of marker migration in this group of patients may be attributed to removing the prostate capsule during prostatectomy surgery. Nevertheless, the ultimate impact on treatment planning decisions was negligible.

In these cases, the IGRT planning was performed based on the other two markers without disruption. The significant advantage of this study is the inclusion of patients after radical prostatectomy. This study demonstrated that inserting a transrectal FM in patients with a history of radical prostatectomy is safe and successful when using anatomical markers like the vesicourethral anastomosis site. Although the risk of complications like sepsis is slightly higher than in non-surgical patients, it remains relatively low. Additionally, the risk of marker migration is also negligible.

## Conclusions

This study, in concert with the literature, demonstrated the feasibility, safety, and reliability of FM insertion for prostate IGRT. This study reported a unicentric experience on FM implantation for prostate IGRT. Given lower complications (compared with previous series), our approach can serve as a basis for clinical practice and future studies. The study findings demonstrated that using narrow needles (18-gauge) can reduce the immediate complications, such as urinary retention and pelvic hematoma. Besides, appropriate bowel preparation and irrigation with povidone-iodine in accordance with broad-spectrum antibiotics can significantly reduce the sepsis rates. Generally, TRUS-guided implantation of FMs is safe and well tolerated in the majority of patients. Migration of FMs is very rare and when it does occur, the effect is negligible. The use of three markers provides a tool to monitor prostate position and volume changes and there is convincing evidence that this procedure is safe for image guided radiotherapy and it is highly recommended.

## Data Availability

The datasets generated and analyzed during the current study are not publicly available due to the restrictions of not obtaining relevant consent from patients. However, they are available from the corresponding author upon reasonable request.

## Declarations

### Confirmation Statement

The authors primarily involved in education or medical research and not directly supported by the government.

## References

[1] Sung H, Ferlay J, Siegel RL, Laversanne M, Soerjomataram I, Jemal A (2021). Global Cancer Statistics 2020: GLOBOCAN estimates of incidence and Mortality Worldwide for 36 cancers in 185 countries. Cancer J Clin.

[2] Houshyari M, Mofid B, Alavi Tabatabaee M, Bakhshandeh M, Taghizadeh-Hesary F (2022). Acute toxicity of 4-week versus 5-week hypofractionated radiotherapy in localised prostate cancer. I Radiother Pract.

[3] Pepe P, Pennisi M (2022). Should 68Ga-PSMA PET/CT replace CT and bone scan in clinical staging of high-risk prostate Cancer?. Anticancer Res.

[4] Juneja P, Kneebone A, Booth JT, Thwaites DI, Kaur R, Colvill E (2015). Prostate motion during radiotherapy of prostate cancer patients with and without application of a hydrogel spacer: a comparative study. Radiat Oncol.

[5] Kron T, Thomas J, Fox C, Thompson A, Owen R, Herschtal A (2010). Intra-fraction prostate displacement in radiotherapy estimated from pre- and post-treatment imaging of patients with implanted fiducial markers. Radiother Oncol.

[6] Schallenkamp JM, Herman MG, Kruse JJ, Pisansky TM (2005). Prostate position relative to pelvic bony anatomy based on intraprostatic gold markers and electronic portal imaging. Int J Radiat Oncol Biol Phys.

[7] De Cicco L, Bracelli S (2019). Fiducial markers implantation for prostate image-guided radiotherapy: a report on the transperineal approach. Radiol Med.

[8] Schiffner DC, Gottschalk AR, Lometti M, Aubin M, Pouliot J, Speight J (2007). Daily electronic portal imaging of implanted gold seed fiducials in patients undergoing radiotherapy after radical prostatectomy. Int J Radiat Oncol Biol Phys.

[9] Pepe P, Tamburo M, Pennisi M, Marletta D, Marletta F (2021). Clinical outcomes of Hydrogel Spacer Injection Space OAR in Men submitted to Hypofractionated Radiotherapy for prostate Cancer. In Vivo.

[10] Gill S, Li J, Thomas J, Bressel M, Thursky K, Styles C (2012). Patient-reported complications from fiducial marker implantation for prostate image-guided radiotherapy. Br J Radiol.

[11] Igdem S, Akpinar H, Alço G, Agaçayak F, Turkan S, Okkan S (2009). Implantation of fiducial markers for image guidance in prostate radiotherapy: patient-reported toxicity. Br J Radiol.

[12] Linden RA, Weiner PR, Gomella LG, Dicker AP, Suh DB, Trabulsi EJ (2009). Technique of outpatient placement of intraprostatic fiducial markers before external beam radiotherapy. Urology.

[13] Shinohara K, Roach M (2008). 3rd. Technique for implantation of fiducial markers in the prostate. Urology.

[14] Moman MR, van der Heide UA, Kotte ANTJ, van Moorselaar RJA, Bol GH, Franken SPG (2010). Long-term experience with transrectal and transperineal implantations of fiducial gold markers in the prostate for position verification in external beam radiotherapy; feasibility, toxicity and quality of life. Radiother Oncol.

[15] Loh J, Baker K, Sridharan S, Greer P, Wratten C, Capp A (2015). Infections after fiducial marker implantation for prostate radiotherapy: are we underestimating the risks?. Radiat Oncol.

[16] Kably I, Bordegaray M, Shah K, Salsamendi J, Narayanan G (2014). Single-center experience in prostate fiducial marker placement: technique and midterm follow-up. J Vasc Interv Radiol.

[17] Langenhuijsen JF, van Lin EN, Kiemeney LA, van der Vight LP, McColl GM, Visser AG (2007). Ultrasound-guided transrectal implantation of gold markers for prostate localization during external beam radiotherapy: complication rate and risk factors. Int J Radiat Oncol Biol Phys.

[18] Dundee P, Furrer MA, Corcoran NM, Peters J, Pan H, Ballok Z (2022). Defining Prostatic Vascular Pedicle recurrence and the anatomy of local recurrence of prostate Cancer on prostate-specific membrane Antigen Positron Emission Tomography/Computed Tomography. Eur Urol Open Sci.

[19] Nguyen DP, Giannarini G, Seiler R, Schiller R, Thoeny HC, Thalmann GN (2013). Local recurrence after retropubic radical prostatectomy for prostate cancer does not exclusively occur at the anastomotic site. BJU Int.

[20] Sihono DSK, Ehmann M, Heitmann S, von Swietochowski S, Grimm M, Boda-Heggemann J (2018). Determination of intrafraction prostate motion during external beam radiation therapy with a transperineal 4-dimensional ultrasound real-time tracking system. Int J Radiation Oncology* Biology* Phys.

[21] Siavashpour Z, Taghizadeh-Hesary F, Rakhsha A (2020). Recommendations on management of locally advanced rectal cancer during the COVID-19 pandemic: an iranian consensus. J Gastrointest cancer.

[22] Escudero JUJ, Peidro JP, de Campos MR, Torrecilla JL, Alcina EL, Verdejo PN et al. Insertion of intraprostate gold fiducial markers in prostate cancer treatment. 2010.

[23] Kok D, Gill S, Bressel M, Byrne K, Kron T, Fox C (2013). Late toxicity and biochemical control in 554 prostate cancer patients treated with and without dose escalated image guided radiotherapy. Radiother Oncol.

[24] Singh J, Greer PB, White MA, Parker J, Patterson J, Tang CI (2013). Treatment-related morbidity in prostate cancer: a comparison of 3-dimensional conformal radiation therapy with and without image guidance using implanted fiducial markers. Int J Radiat Oncol Biol Phys.

[25] Sveistrup J, af Rosenschöld PM, Deasy JO, Oh JH, Pommer T, Petersen PM (2014). Improvement in toxicity in high risk prostate cancer patients treated with image-guided intensity-modulated radiotherapy compared to 3D conformal radiotherapy without daily image guidance. Radiat Oncol.

[26] Moman MR, van der Heide UA, Kotte AN, van Moorselaar RJ, Bol GH, Franken SP (2010). Long-term experience with transrectal and transperineal implantations of fiducial gold markers in the prostate for position verification in external beam radiotherapy; feasibility, toxicity and quality of life. Radiother Oncol.

[27] Crook JM, Raymond Y, Salhani D, Yang H, Esche B (1995). Prostate motion during standard radiotherapy as assessed by fiducial markers. Radiother Oncol.

[28] Herman MG, Pisansky TM, Kruse JJ, Prisciandaro JI, Davis BJ, King BF (2003). Technical aspects of daily online positioning of the prostate for three-dimensional conformal radiotherapy using an electronic portal imaging device. Int J Radiat Oncol Biol Phys.

[29] Serago CF, Buskirk SJ, Igel TC, Gale AA, Serago NE, Earle JD (2006). Comparison of daily megavoltage electronic portal imaging or kilovoltage imaging with marker seeds to ultrasound imaging or skin marks for prostate localization and treatment positioning in patients with prostate cancer. Int J Radiat Oncol Biol Phys.

[30] van der Heide UA, Kotte AN, Dehnad H, Hofman P, Lagenijk JJ, van Vulpen M (2007). Analysis of fiducial marker-based position verification in the external beam radiotherapy of patients with prostate cancer. Radiother Oncol.

[31] de Boer J, de Bois J, van Herk M, Sonke JJ (2012). Influence of the number of elongated fiducial markers on the localization accuracy of the prostate. Phys Med Biol.

[32] Pouliot J, Aubin M, Langen KM, Liu YM, Pickett B, Shinohara K (2003). Non)-migration of radiopaque markers used for on-line localization of the prostate with an electronic portal imaging device. Int J Radiat Oncol Biol Phys.

[33] Kupelian PA, Willoughby TR, Meeks SL, Forbes A, Wagner T, Maach M (2005). Intraprostatic fiducials for localization of the prostate gland: monitoring intermarker distances during radiation therapy to test for marker stability. Int J Radiat Oncol Biol Phys.

[34] Poggi MM, Gant DA, Sewchand W, Warlick WB (2003). Marker seed migration in prostate localization. Int J Radiat Oncol Biol Phys.

